# Taiwan National Health Insurance and the Difference between Proportional Physician Fee of Outpatient and Inpatient Ward in General Hospital during the COVID-19 pandemic : Case Report

**DOI:** 10.1192/j.eurpsy.2023.691

**Published:** 2023-07-19

**Authors:** S.-C. Wang, Y.-H. Lin

**Affiliations:** Tao Yuan General Hospital, Ministry of Health and Welfare, Taiwan, Taoyuan City, Taiwan, Province of China

## Abstract

**Introduction:**

According to CEOWORLD Magazine‘s 2019 “Health Care Indicators” rating of 89 countries in the world, Taiwan ranks first in the world. The coverage of the National Health Insurance covers all necessary medical treatment, including outpatient, hospitalization, and prescription drugs etc. The psychiatrist was wondering which item with the highest service cost–performance (CP) ratio of the psychiatrist performance in a general hospital and used proportion of PPF as performance indicator. He used allocation to distribute the hours across job activities.

**Objectives:**

The purpose of this study is to investigate items allocation proportion for outpatient and inpatient ward in a specific month, examining the distribution of performance and figure out an appropriate model to optimal medical service.

**Methods:**

Demographic data were collected through PPF projects included 15 outpatient items and 19 inpatient ward items from the third-month of the psychiatrist’s employment in the general hospital, as shown in **Table 1**. Items related to physiological examination has been excluded. The performance is calculated by combining outpatient and inpatient wards.

**Results:**

Demographic data analysis found that proportion of inpatient ward PPF (67.01%) was significantly greater than proportion of outpatient PPF (32.99%) **(Figure 1)**. The inpatient ward performance was 2 times the outpatient performance. This result showed that most performance came from inpatient ward. Among all items of the proportion of PPF unit, the highest two for inpatient ward items were general hospital bed inpatient consultation (32.58%) and special treatment for psychiatric inpatients (14.35%), and for outpatient, the highest was psychiatric outpatient consultation - more than two (11.31%) **(Table 1)**.

**Image:**

**Figure:**
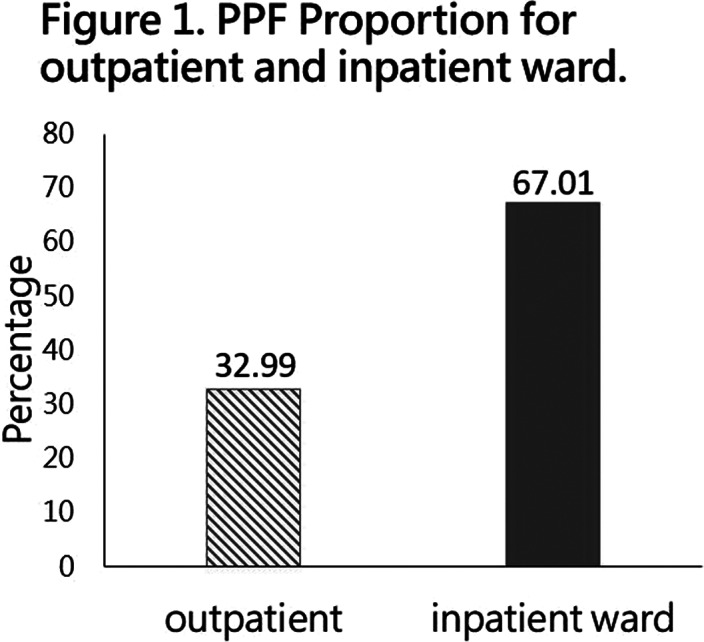

**Image 2:**

**Figure:**
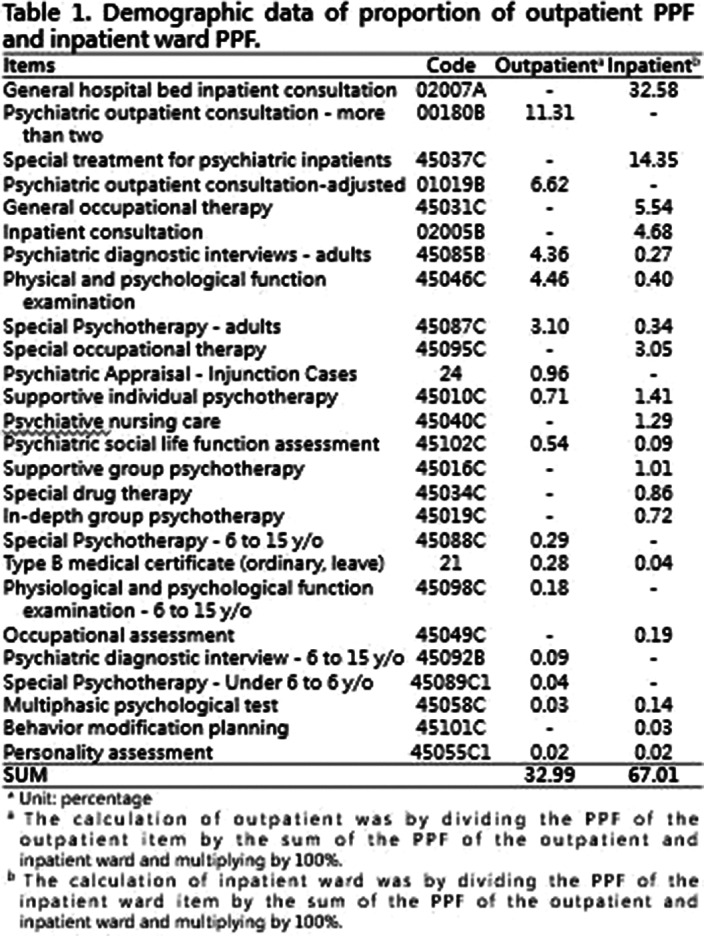

**Conclusions:**

National Health Insurance is an important system to assist in epidemic prevention during the COVID-19 period. The most PPF of psychiatrist service in a general hospital came from the service of inpatient ward items, including the general hospital bed inpatient consultation with the highest CP ratio. These findings may suggest that the inpatient service could offer psychiatrists fair PPF, and the hospital environment could be favorable for recruitment.

**Disclosure of Interest:**

None Declared

